# Ergot of Rye as an Oxytocic

**Published:** 1894-09

**Authors:** J. G. Swayne

**Affiliations:** Professor of Midwifery in University College, Bristol, and Consulting Physician Accoucheur to the Bristol General Hospital


### ERGOT OF RYE AS AN OXYTOCIC.

BY

J. G. Swayne, M.D. Lond.,

*Professor of Midwifery in University College, Bristol, and Consulting Physician Accoucheur to the Bristol General Hospital.*

---

Ergot of rye is a remedy whose powers as an oxytocic, or accelerator of difficult labour, were formerly so much believed in and over-praised, especially in Great Britain, that German writers used to call it, in derision, the English accoucheur's "Herr Gott." Of late years, however, it has, in this respect, gone very much out of fashion and out of favour, and is now seldom employed until *after* labour in order to check hemorrhage. This disuse of ergot *during* labour has arisen principally from two causes. The first of these is that when given before delivery, as it was formerly, it was not only used, but also very much abused. For instance, ignorant and incompetent practitioners were frequently in the habit of giving it to hasten tedious labour that was occasioned solely by the presence of some mechanical obstacle. In this way they invoked and called to their aid the powers, as Dr. Barnes has remarked, of a Frankenstein, quite beyond their control, whose unbridled strength might give rise to frightful disasters; in their ignorance they quite forgot that the proper and legitimate use of this drug, *during* labour, is to stimulate, strengthen and sustain deficient powers of the uterus, but only, however, when the retarded labour is due to this cause and no other. The second cause of the disuse of ergot during labour is that in the present day obstetric practitioners have learned that in the forceps they have an ally that is much safer and more efficient, because it is completely under their own control. There are some exceptional cases, however, especially those of simple deficiency of uterine action, in which ergot supplies this natural deficiency, and produces much better after-effects than the use of the forceps does. I attended very lately a case of this kind in which employed ergot with the happiest results, and its effects were so admirable that I think them worth recording, if only to show what a valuable birth-help we may sometimes inadvertently neglect.

I attended, on the 13th of last February, a lady about 25 years of age, who had borne three children previously. She was a blonde of healthy appearance and good physique; but I had not attended her at her former confinements. She told me that the first was not tedious, and was on the whole favourable. It happened seven years ago, and the child, a girl, is now living. The second labour began with rupture of the membranes, and was very tedious, from want of proper pains. It ended in the birth of a stillborn male child. In the third labour the medical man in attendance used the forceps. The brow was much compressed, in consequence, I imagine, of an occipito-posterior position. The child, a boy, was delicate from its birth, and died about 11 months after. On the present occasion I was first sent for to see her on Saturday morning, February 10th, 1894. On arriving at the house I was told that the waters had escaped early in the morning, but that there had been no pains of any consequence since. On examining I found scarcely any dilatation of the os uteri, which was, however, soft and cushiony, but yet would not admit more than the tip of the index finger. The child's head, which could not be reached by an ordinary examination, lay rather forward above the os pubis, but it could be felt through the anterior lip of the os uteri as if lying in the ordinary position. As there was no show or any other sign of impending labour, I left the house, and called again in the afternoon, to find everything in just the same condition as in the morning. I did not therefore feel inclined to stay; but the patient's mother-in-law, who was evidently a very intelligent person and had been with her in all her former labours, begged me to sleep there that night, as their house was distant a mile and a half from mine. She informed me that the second labour, which began in a precisely similar manner, came on quickly without any warning in the night, and was over before medical aid could be procured, the child being stillborn. I therefore agreed to stay there. In the following morning, however, I found that there had been no labour pains, and that there was no alteration in the shape of the os uteri. I therefore went home; but I called again in the evening to see her, and finding everything in the same condition, I did not stop there that night. On the following day, Monday, February 12th, I received a note in the morning asking me to call as soon as I could, because my patient had felt no movements of the child for several hours. On arriving at the house, about 10.30 a.m., I found that I could detect no fœtal movements with the hand, but on listening with the stethoscope I thought that I could hear feeble cardiac and placental sounds, but was not quite sure of this, and on examining the napkins, I observed that the liquor amnii, which was still flowing away, was stained with meconium. The os uteri was in just the same undilated condition and would not readily admit the tips of two fingers, and there had been no pains worthy of the name.

The lingering labour had evidently compromised the safety of the child. On mentioning my apprehensions to the mother-in-law of the lady, she anxiously inquired if, without risk to the mother, I could save the child's life by any operation that would insure prompt delivery? I told her that, in the complete absence of dilating pains, I should have to forcibly dilate the parts by my hand before I could deliver by turning the child, or using the forceps with sufficient promptitude to effect that object; whereas such operations, when performed so hurriedly, could not fail to be attended with risk to both mother and child. I added, however, that we had a good old-fashioned remedy, called Ergot of Rye, for inducing prompt uterine action, which, although sometimes uncertain in its action, and disappointing in its results when not preceded by distinct and decided uterine contractions, was under present circumstances the best thing to give, as, if it were successful, it would ensure delivery with more promptitude and safety than by employing any other means. I accordingly gave her, at about 11 a.m., twenty minims of the fluid extract of ergot of the American Pharmacopœia, which I have long used and find more dependable than our own. I stayed with her half an hour to observe its effects. I kept one hand on her abdomen all the time, and about twenty minutes after she had taken the ergot I felt a distinct uterine contraction, of which, although it was not painful, she was sensible, because she felt that it expelled some more liquor amnii. At the end of the half-hour I gave her a similar dose, and directed another one to be given half an hour later. I then went away for a short time, having previously left directions that I should be sent for as soon as strong regular pains had set in. About 2.30 p.m. I received a message that I was wanted immediately, and on repairing to the house without delay, the first sound I heard was my patient crying out for chloroform. On examining I found the os uteri almost fully dilated, and strong forcing pains coming on at regular intervals. As I was informed that, at her last labour, she had had a rather dangerous amount of *post-partum* hemorrhage, I again gave her ʒi of the extract of ergot, and after a dozen more pains the child, a fine boy, was expelled. It was born at exactly a quarter to four p.m. At first it made no respiratory efforts, although the umbilical arteries were still pulsating feebly. In consequence of what I had heard about the previous labour, I wished to keep up continuous pressure on the uterus until the placenta was expelled; so I did not separate the child, but requested the father, who was present, to rub it briskly with warm flannels, to douche it alternately with cold and warm water, and perform artificial respiration by the Silvester method, which he did very well, after I had given him previous instructions. In about a quarter of an hour after the birth of the child, the placenta was expelled by expression, together with about a pint of blood. This, however, caused no unpleasant symptoms, and there was no further hemorrhage; but I kept up pressure on the uterus for about an hour after delivery. As to the child, after continuing artificial respiration for more than half an hour, we observed that it began to breathe, and at last heard it cry lustily, to the great joy of its father and mother, who were exceedingly anxious to have a son, having lost two previously. Everything then went on smoothly and ended as well as possible for both mother and child.

The chief feature in this case was that the pains induced by ergot of rye bore an exact resemblance to the natural pains which we meet with in ordinary unassisted labour. And yet there is no reason to doubt, I think, that these pains were the direct result of giving ergot; for the first one came on just within half an hour after the first dose was given, and the pains then continued uninterruptedly, and gradually increasing in force, until the child was born within five hours afterwards; and I had given the first dose at a time when there was a complete absence of pains of any kind. In this last respect the action of ergot in the present case appeared to differ from what is ordinarily observed. Many authorities think that, although it is capable of increasing uterine pains which already exist, it is incapable of exciting them *de novo* as it undoubtedly did in my own case just related. In this case I also transgressed an ordinary rule of practice, which is, not to give ergot until the first stage is over; whereas I began to give it when the os uteri would scarcely admit the extremities of two fingers; and this non-observance of a common rule not only did no harm but was followed by the best possible results. I can easily imagine that in a primipara when delay in the first stage of labour arises from the obstacle presented by a rigid os uteri, the use of ergot might cause serious injury; but not so in a multipara with a soft dilatable os, as in my case. It cannot, however, be overlooked that in this case, also, the action of ergot was so much more favourable, and the pains more resembling the natural pains, than they usually are. For instance, Dr. Lauder Brunton states that "ergot causes contraction of the uterus, especially of the pregnant uterus. The contraction is not usually so much rhythmical as *tetanic* in nature, and occasionally increases in violence. There is no complete relaxation between the spasms, as in the ordinary labour pains." But in my case the quiescent intervals between the pains were very regular and well marked. The child's life hung upon such a thread that I am confident that it would have died if the pains had assumed the *tetanic* character above described, so as to interfere with the utero-placental circulation; or even if ergot had exerted that kind of toxic influence on the child, which is often attributed to it. With respect to the mother, I feel sure that if, instead of giving ergot, I had rapidly delivered by turning or the forceps, that the sudden emptying of a flabby uterus would have caused serious *post-partum* hemorrhage, and also increased danger to the infant. On the whole I consider that I was very fortunate in having to deal with a patient upon whom, perhaps, from some peculiar idiosyncrasy, ergot of rye acted so kindly and beneficially.

